# Microstructure Evolution and Deformation Behavior of Extruded Mg-5Al-0.6Sc Alloy during Room and Elevated Temperature Tension Revealed by Ex-Situ EBSD and VPSC

**DOI:** 10.3390/ma16134534

**Published:** 2023-06-22

**Authors:** Lei Zhang, Shiyu Luan, Shuai Yuan, Jinhui Wang, Lijia Chen, Peipeng Jin

**Affiliations:** 1 School of Material Science and Engineering, Shenyang University of Technology, Shenyang 110870, China; 2 Qinghai Provincial Key Laboratory of New Light Alloys, Qinghai Provincial Engineering Research Center of High Performance Light Metal Alloys and Forming, Qinghai University, Xining 810016, China

**Keywords:** Mg-5Al-0.6Sc alloy, EBSD, VPSC, microstructure evolution, deformation behavior

## Abstract

In this study, the microstructure evolution and deformation behavior of the extruded Mg-5Al-0.6Sc (AS51) alloy during tensile testing at room temperature (RT) and 250 °C were investigated by electron backscattered diffraction (EBSD) characterization and Visco Plastic Self Consistent (VPSC) simulation. The results showed that a continuous hardening behavior of the alloy occurred during the deformation at RT, and a certain softening was caused by the occurrence of dynamic recovery (DRV) and dynamic recrystallization (DRX) in the late stage of deformation at 250 °C. The primary deformation mechanism at both RT and 250 °C was dislocation slip, with prismatic <a> slip being the dominant deformation mode, and no significant changes in grain size or texture type occurred. By identifying the activated twin variants, the results indicated that the selection of twin variants was closely related to the local stress concentration. The relatively low activation frequency of extension twinning at 250 °C is partly attributed to the fact that the consumption of dislocations by DRV and DRX can effectively relax the local stress concentration. Meanwhile, the DRX mechanism during the deformation of the alloy at 250 °C was mainly discontinuous dynamic recrystallization (DDRX), with a low recrystallization fraction.

## 1. Introduction

Magnesium (Mg) alloys have the competitiveness of high specific strength and specific stiffness, excellent damping properties, low density, and outstanding biocompatibility, so they are widely used in defense and military, biomedical, and aerospace fields. Unfortunately, the poor plastic deformability and low absolute strength of Mg alloys at RT greatly hinder the further application of Mg alloys in the field of structural materials [1,2]. To facilitate the development and application of Mg alloy materials, researchers have made numerous explorations for this purpose. From alloy composition optimization, structural design, processing technology, heat treatment, and other aspects of improving the performance of Mg alloys have been systematically investigated [3,4,5].

Presently, the Mg alloys that have achieved some scales of commercial application are predominantly Mg-Al alloys. The second phase is an effective means of strengthening Mg alloys [6,7]. However, the major second phase in Mg-Al alloys, Mg_17_Al_12_, has a low melting point. When the service temperature is high, the strength of Mg-Al alloys decreases dramatically, which greatly limits the application of Mg-Al alloys. The main mechanism of dislocation slip in Mg alloys at RT is basal <a> slip, and Mg_17_Al_12_, as a disc-shaped precipitated phase parallel to the (0001) of α-Mg cannot effectively pin the motion of basal dislocations [8], so the strengthening effect of Mg_17_Al_12_ at RT is also limited. Thus, the introduction of other high-melting point and high-modulus second phases by alloying is an effective strategy to further improve the mechanical properties of Mg-Al alloys. It was found that the addition of rare earth (RE: Gd, La, Ce, Nd, Sc, etc.) to Mg-Al alloys could not only produce strong solid solution strengthening but also form high-strength Al-RE intermetallic compounds [3,9], which could effectively enhance the mechanical properties of Mg alloys both at RT and elevated temperature (ET).

The deficiency of plastic deformability of Mg alloys at RT originates from the hexagonal close-packed (HCP) structure of Mg with a c/a value of 1.624, and this low symmetry structure leads to a much higher critical resolved shear stress (CRSS) in the non-basal slip system than in the basal slip system at RT [10]. Therefore, the non-basal slip system is difficult to be activated during RT deformation and cannot effectively coordinate the stress along the c-axis to achieve uniform plastic deformation [11,12]. In contrast, twinning, as a deformation mode that can coordinate the c-axis strain, has a strong orientation dependence [13]. Concurrently, the strain that can be directly contributed by twinning is small, and the twin boundaries or twin boundary intersections usually lead to stress concentration due to dislocation accumulation and become potential crack-forming nucleation sites [14,15]. As a consequence, Mg alloys generally exhibit poor RT plasticity. It is suggested that adding RE to Mg can weaken the anisotropy and reduce the CRSS ratio of non-basal and basal slip and the cross-slip barrier [2,5]. This promotes the activity of the non-basal slip system, which provides more pathways for dislocation slip and allows the plasticity of the Mg alloy to be improved. Therefore, Mg alloys containing RE are currently receiving widespread attention [16,17]. Compared to other RE, Sc has superior grain refinement effects, high solubility (>25 wt.%) in Mg, and can significantly improve the corrosion resistance of Mg alloys, which means that Sc-containing Mg alloys have great potential for applications [18].

The CRSS of non-basal slip is significantly reduced during the ET deformation of Mg alloys, allowing a large activation of non-basal slip to yield a uniform plastic deformation [19]. Therefore, Mg alloys usually have good plastic deformation ability at ET deformation conditions. In addition, for fine grain Mg alloys (grain size < 10 μm), grain boundary sliding provides a possible way for Mg alloys to achieve excellent plasticity and even superplasticity [11]. Although the initiation of multiple deformation modes during ET deformation can effectively coordinate the deformation of polycrystalline Mg alloys, the consumption of dislocations by DRV and DRX can also reduce the stress concentration inside the material to some extent [19,20], thus diminishing the potential crack nucleation sites. It is critical to investigate the deformation mechanism and microstructure evolution during the ET deformation of Mg alloys to develop Mg alloys with superior mechanical properties at ET.

Therefore, in this study, the extruded AS51 alloy was used as the object of study to characterize the microstructure at different strains at RT and 250 °C. The microstructure evolution of the alloy during deformation at RT and ET was revealed. The deformation mechanisms of the alloy at RT and ET were examined in detail by combining VPSC simulations and the obtained EBSD data. The results contribute to further understanding the RT and ET deformation behavior and microstructure evolution of Mg alloys.

## 2. Materials and Methods

For the preparation of as-cast AS51 alloys, one can refer to our previous report [21]. The alloy ingots obtained from melting and casting are machined into ϕ44 × 44 mm cylindrical billets by lathe, and then the billets are homogenized at 420 °C for 8 h before hot extrusion. The surface of the extrusion die is coated with graphite powder for lubrication. Subsequently, the extrusion die, the billets and the extrusion indenter are preheated at 450 °C for 1 h to achieve a uniform temperature. The preheated billets were extruded by a four-column hydraulic press (Shandong Tengzhou Zhonghe Machinery Factory, Tengzhou, China) with an extrusion temperature of 450 °C and an extrusion ratio of 13:1, then air-cooled to RT. The diameter of the obtained extruded rod was 12 mm. A bone-shaped specimen is cut on an extruded rod by electric discharge machining with a gauge of 15 mm (L) × 4 mm (W) × 2 mm (T). The Instron-5982 universal testing machine was used to perform tensile properties testing of the extruded alloys at RT and 250 °C, with the loading direction parallel to the extrusion direction (ED) and a tensile rate of 0.1 mm/min. To perform a hot tensile test at 250 °C, the heating rate was set to 5 °C/s. After heating up to 250 °C, the tensile test was started after holding for 300 s. The samples were water-cooled immediately after the hot tensile test to preserve the deformed microstructure. EBSD characterization was performed on the centers of specimens with engineering strains of 5%, 10%, and 15% at RT deformation conditions. The microstructure at the center of the specimens deformed to strains of 5%, 10%, 15%, and 20% at 250 °C was characterized by EBSD. The test was stopped when the target strain was achieved during tensile deformation, and then the corresponding sample was taken for microstructure characterization. The microstructure was characterized by a Zeiss scanning electron microscope equipped with an EBSD detector. SEM and EBSD test samples were prepared using mechanical polishing combined with electrolytic polishing, and EBSD data processing was performed using the Aztec Crystal.

## 3. Results

### 3.1. Initial Microstructure

**Figure 1:**
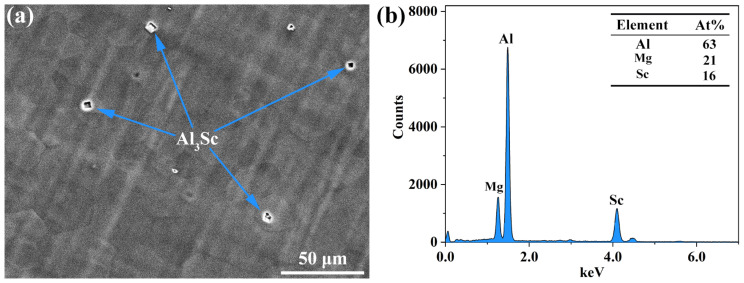
(**a**) SEM image of the extruded AS51 alloy, (**b**) EDS results of the second phase.

**Figure 2:**
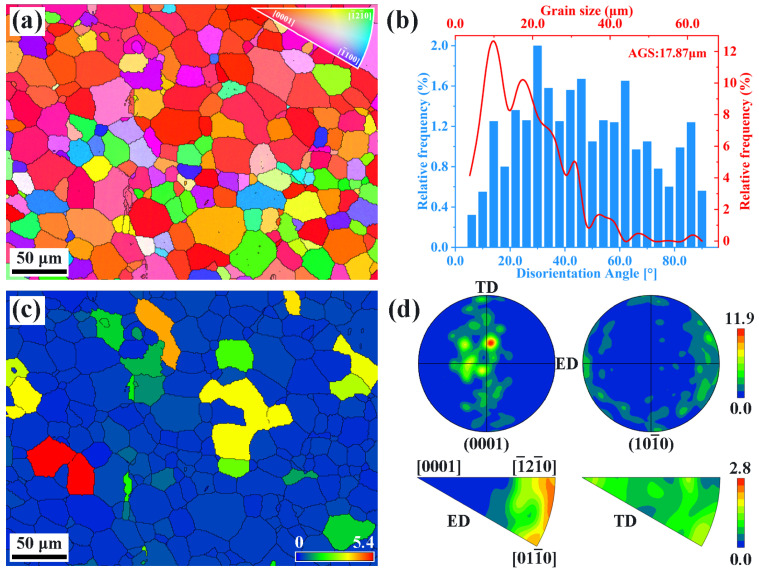
(**a**) IPF map, (**b**) grain size and misorientation angle distribution figure, (**c**) GOS map, and (**d**) PF and inverse PF of the extruded AS51 alloy.

Figure 1a shows the SEM image of the extruded AS51 alloy, and Figure 1b presents the EDS results of the second phase in Figure 1a, indicating that the bulk second phase in the alloy is Al_3_Sc. Figure 2a shows the IPF map of the extruded AS51 alloy, which is mainly composed of equiaxed grains. The grain size distribution is shown in Figure 2b, and the average grain size is 17.87 μm. The misorientation angle distribution map shown in Figure 2b illustrates that the grain boundaries in the alloy are mainly high-angle grain boundaries (HAGBs), and combined with the grain orientation spread (GOS) map shown in Figure 2c, it can be determined that the grains in the extruded alloy are mainly recrystallized grains. According to the pole figure (PF) and the inverse PF shown in Figure 2d, the extruded alloy exhibits a basal fiber texture of (0001)//ED with a maximum relative texture intensity of 11.9.

### 3.2. Engineering Stress–Strain Curve

**Figure 3:**
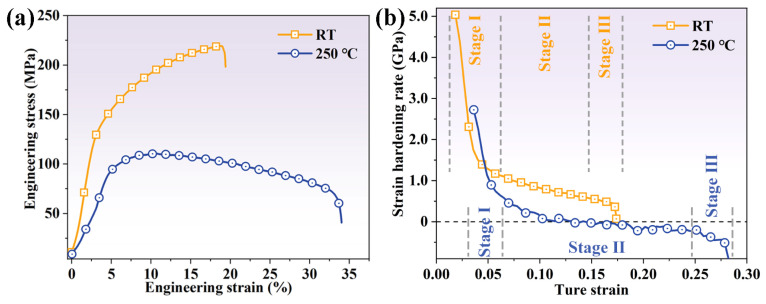
(**a**) Engineering stress–strain curves of the extruded AS51 alloy at RT and 250 °C, and (**b**) corresponding strain hardening curves.

Figure 3a illustrates the engineering stress–strain curves of the extruded AS51 alloy at RT and 250 °C. The tensile yield strength (TYS), ultimate tensile strength (UTS), and elongation (EL) of the alloy at RT are 122.7 MPa, 219.3 MPa, and 19.7%, respectively. The TYS, UTS, and EL of the alloy at 250 °C are 89.2 MPa, 110.3 MPa, and 33.2%, respectively. Compared to RT deformation, TYS and UTS are reduced by 27.3% and 49.7%, respectively, while EL is improved by 68.5%. The strain hardening curves shown in Figure 3b at both RT and 250 °C present a monotonically decreasing feature, which indicates that the extruded AS51 alloy has no significant extension twinning activity during deformation. The strain hardening curve at RT shows the three stages of deformation of the alloy. Stage I is the rapidly decreasing stage, corresponding to the elastic–plastic transition stage; stage II is the slowly decreasing stage, corresponding to the linearly decreasing strain hardening behavior; stage III is the rapidly decreasing strain hardening rate until fracture [22,23]. The trend of the strain hardening curve at 250 °C is approximately the same as that at RT, with the difference that the work hardening rate gradually decreases to negative values after a true strain of 0.1 when deformed at 250 °C, indicating that the softening effect caused by DRV and DRX occurs [20].

### 3.3. Microstructure Evolution

**Figure 4:**
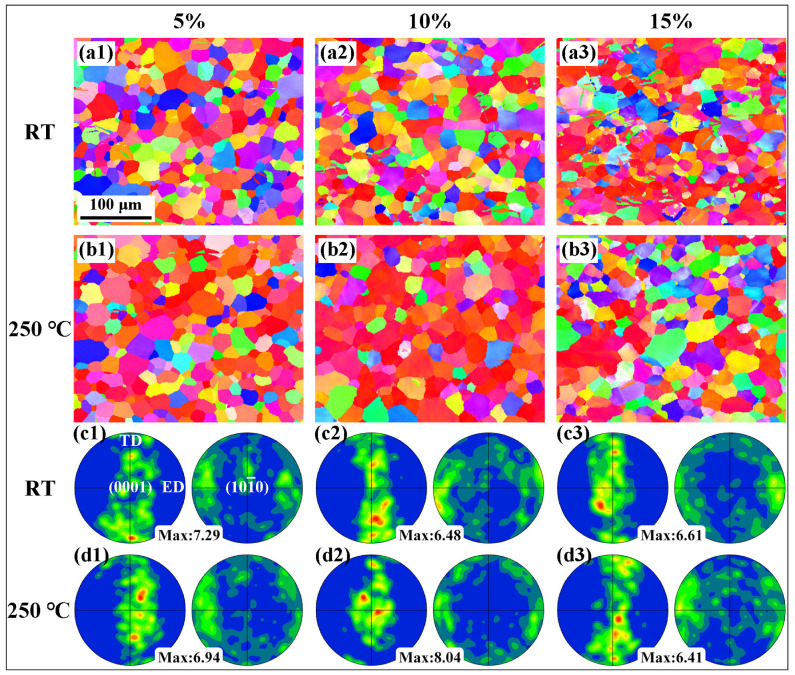
IPF maps and PFs of the extruded AS51 alloy at different strains at RT and 250 °C.

Figure 4(a1–a3) shows the IPF maps of the extruded AS51 alloy at RT at strains of 5%, 10%, and 15%, respectively. The twin content in the microstructure of the alloy gradually increases with increasing strain. The color gradient inside the grain gradually becomes noticeable, indicating that the increase in strain enhances the intragranular slip activity [24]. Figure 4(b1–b3) shows the IPF maps of the alloy at different strains at 250 °C. Unlike the deformed microstructure at RT, almost no twinning is observed in the ET deformed structure at strains of 5%, 10%, and 15%, respectively. The PFs at different strains shown in Figure 4(c1–d3) indicate that the type of texture during tensile deformation of the alloy at RT and 250 °C stays the same as that before deformation.

**Figure 5:**
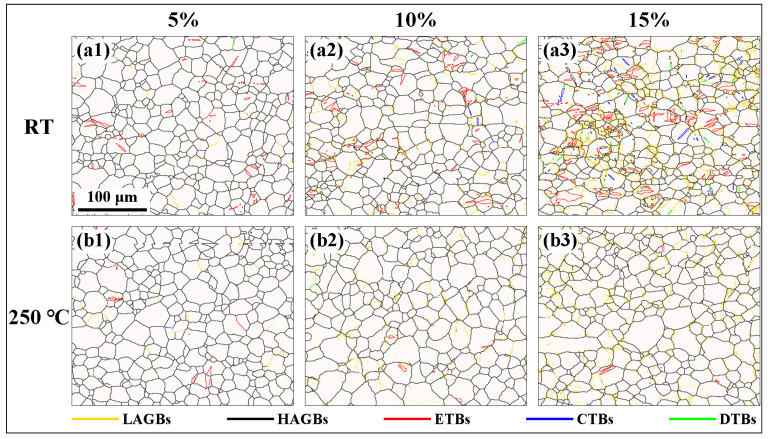
Grain boundary maps of the extruded AS51 alloy at different strains at RT and 250 °C.

Figure 5 shows the grain boundary maps of the extruded AS51 alloy at 5%, 10%, and 15% strain at RT and 250 °C. It can be noticed that the content of low-angle grain boundaries (LAGBs) in the microstructure at RT and ET deformation conditions gradually increases with the increase in strain. LAGBs are composed of a series of dislocations, and grain boundaries can effectively impede dislocation motion [12]. Therefore, as the strain increases, dislocations continuously accumulate near the grain boundaries, leading to a progressively increasing content of LAGBs. In addition, the red lines in Figure 5 indicate the extension twin boundaries (ETBs), the blue lines represent the compression twin boundaries (CTBs), and the green lines denote the double twin boundaries (DTBs). It is further demonstrated that the twin content in the microstructure gradually increases during the RT deformation and is dominated by {10–12} extension twins. In contrast, the twin content in the alloy is extremely low when deformed at ET. This is attributed to the decreased CRSS of non-basal slip during ET deformation [25], which is easily activated, and thus able to coordinate the c-axis strain. Therefore, twinning is no longer the unique mode of deformation to coordinate c-axis strain.

**Figure 6:**
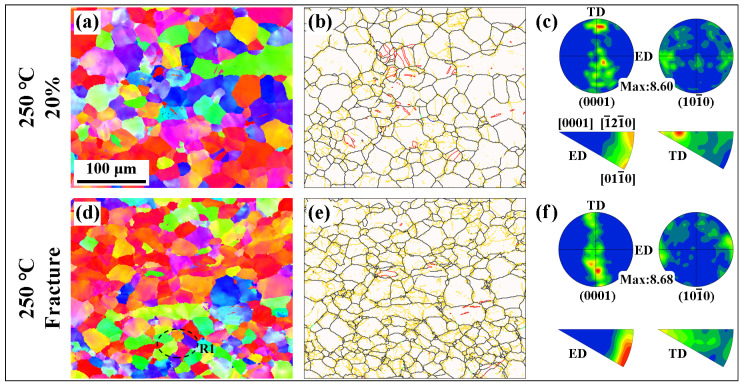
Microstructure of extruded AS51 alloy at 20% strain and near the fracture at 250 °C.

**Figure 7:**
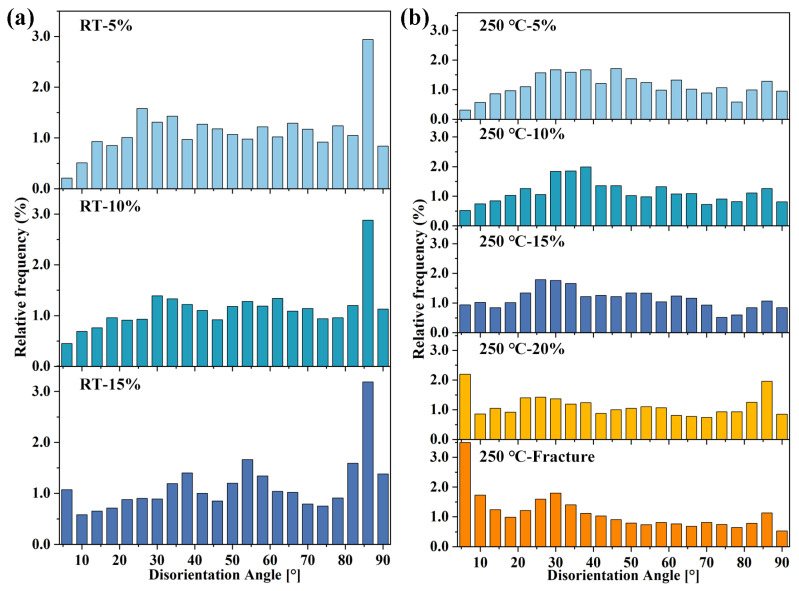
Distribution of misorientation angles of extruded AS51 alloy at different strains at RT (**a**) and 250 °C (**b**).

**Figure 8:**
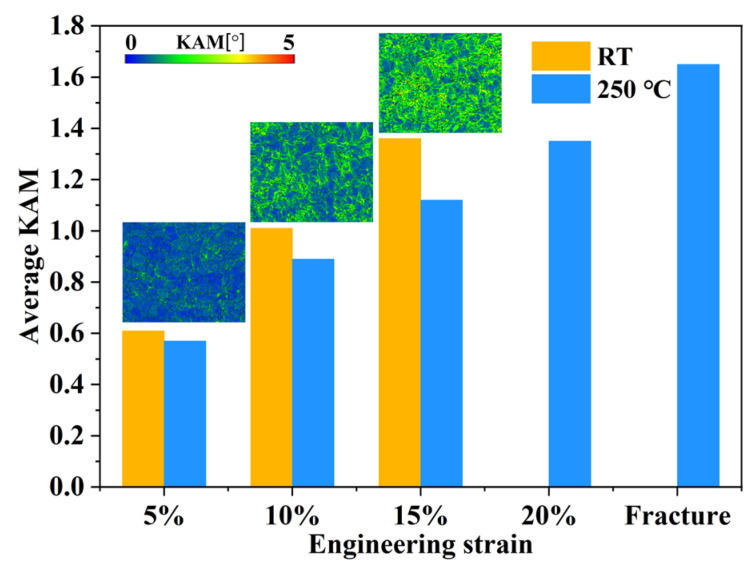
Average KAM of extruded AS51 alloy at different strains at RT and 250 °C. The inset shows the KAM maps at different strains during RT tensile test.

Figure 6 shows the microstructure of the extruded AS51 alloy at 20% strain and near the fracture at 250 °C. As the strain continuously increases, the content of LAGBs in the microstructure after ET deformation increases significantly. Meanwhile, the frequency of twin activation has increased. However, the overall twin content is lower than the microstructure after RT deformation. The distribution of misorientation angles for the extruded AS51 alloy at different strains at RT and 250 °C shown in Figure 7 further demonstrates the variation in LAGBs and twin boundary fractions with strain. Furthermore, the type of alloy texture does not change after ET deformation at high strain, as shown in Figure 6c,f. The average KAM of the extruded AS51 alloy at different strains at RT and 250 °C is statistically presented in Figure 8. The KAM map can reflect the dislocation distribution inside the alloy to a certain extent, and the average KAM can reflect the dislocation density of deformed microstructures [26,27]. The dislocation density increases with increasing strain in both RT and ET deformation microstructures. Additionally, the dislocation density in the RT deformation microstructure is always higher than the corresponding ET deformation microstructure for the same amount of deformation. This can be ascribed to the dislocation consumption by DRV and DRX during ET deformation.

## 4. Discussion

### 4.1. Twinning Behavior

Twinning, as a deformation mechanism that can coordinate c-axis strain during the RT deformation of Mg alloys, has an important role in coordinating the plastic deformation of Mg alloys [28]. Among them, {10–12} extension twinning is the easiest activated twin mode during deformation due to its low CRSS and is widely reported [2,29,30]. As shown in Figure 5, Figure 6 and Figure 7, {10–12} extension twinning is likewise the dominant twinning mode in this study and is significantly activated during the RT deformation of the alloy. The extension twinning Schmid factors (SF) calculated at different strains at RT and 250 °C are listed in Table 1. The results show that for the extension twinning, the SF at different strains at either RT or 250 °C is essentially equal, both being about 0.4. Therefore, the difference in the activation frequency of the extension twin at RT and 250 °C is independent of the SF. In addition, the higher SF of the extension twin facilitates its activation. Meanwhile, the slightly reduced CRSS of twinning at ET also facilitates its initiation. However, the twinning activation frequency of the alloy at 250 °C is very low and the role of twinning in coordinating plastic deformation is limited. Meanwhile, the alloy has a superior plastic deformation ability at 250 °C. Thus, it is further shown that non-basal slip is heavily activated to coordinate the plastic deformation of the alloy during deformation at 250 °C.

**Figure 9:**
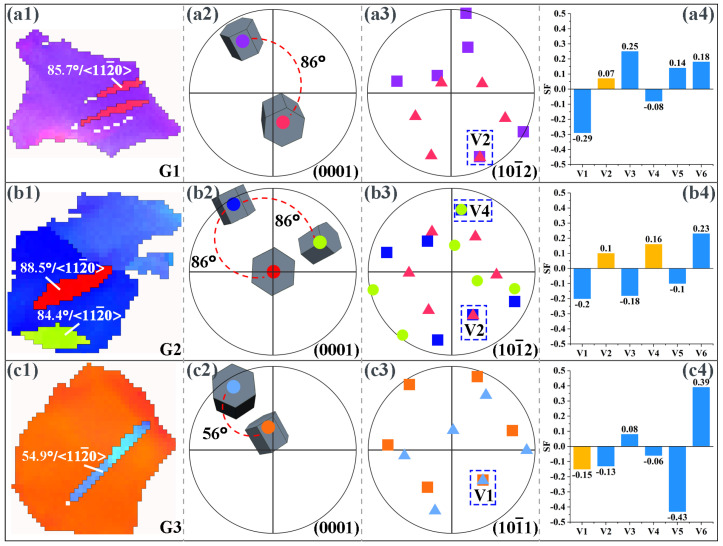
Analysis of twinning variant in the microstructure of extruded AS51 alloy at 15% strain at RT. (**a1**–**c1**) IPF maps, (**a2**–**c2**) (0001) PFs, (**a3**,**b3**) (10–12) PFs, (**c3**) (10–11) PF; (**a4**–**c4**) SF plots of the variants. In all PFs of Figure 9 and Figure 10, colors are used to distinguish where the twins and the original grains are located, and the colors representing the twins and the original grains are consistent with the corresponding IPF maps.

**Figure 10:**
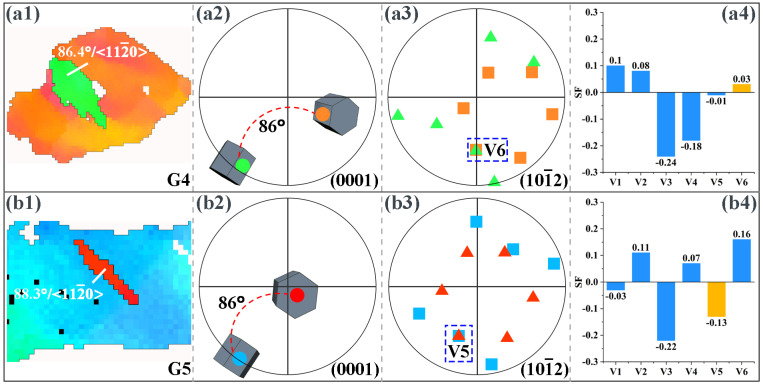
Analysis of twinning variant in the microstructure of extruded AS51 alloy at 20% strain at 250 °C. (**a1**,**b1**) IPF maps, (**a2**,**b2**) (0001) PFs, (**a3**,**b3**) (10–12) PFs, (**a4**,**b4**) SF plots of the variants.

By analyzing the twin variant, it can be determined whether the activation of the twin follows Schmid law or results from stress concentration [13,31]. When a twin is activated, the plane shared by the twin and the original grain is the twinning plane [32,33]. Therefore, for the extension twins and compression twins, the positions where the pole corresponding to the twin coincides with the pole corresponding to the original grain in the (10–12) and (10–11) PFs, respectively, represent that the corresponding twin variant is activated. Figure 9 shows the results of the twin variant analysis in the microstructure of the extruded AS51 alloy at 15% strain at RT. The twins generated in grains G1 and G2 shown in Figure 9(a1,b1) are extension twins with a misorientation angle of about 86° at the twin boundary and a rotation axis of <11–20>. In the corresponding (0001) PF, the relative positions of the twins and the original grains and their 3D cells are shown. In all PFs of Figure 9 and Figure 10, colors are used to distinguish where the twins and the original grains are located, and the colors representing the twins and the original grains are consistent with the corresponding IPF maps. With the (10–12) PF shown in Figure 9, it can be observed that the activated twin variants in grain G1 are V2, and the activated twin variants in grain G2 are V2 and V4. Combining the SFs in Figure 9(a4,b4), the SF of the grain G1 activated twin variant V2 is only 0.07, and the SFs of grain G2 activated twin variants V2 and V4 are 0.1 and 0.16, respectively, neither of which is in accordance with Schmid law. The activated twin in grain G3 shown in Figure 9(c1) is a compression twin, with a misorientation angle of about 54.9° at the twin boundary and a rotation axis of <11–20>. The (10–11) PF in Figure 9(c3) shows that the variant generated by compression twinning in grain G3 is V1 and the corresponding SF is −0.15, again not in accordance with Schmid law. Figure 10 presents the results of twin variant analysis in grains G4 and G5 of the extruded AS51 alloy at a strain of 20% at 250 °C. The twins generated in grains G4 and G5 are extension twins. The extension twin variants activated in grains G4 and G5 are V6 and V5, respectively. The SFs for activating V6 and V5 are 0.03 and −0.13, respectively, ranking fifth and third, respectively, among the SFs for activating the six variants of the extension twin, which also do not follow Schmid law. Therefore, for the tensile deformation process of the extruded AS51 alloy at either RT or 250 °C, the activation of twins does not exactly follow Schmid law. For the five randomly selected grains, the SFs of the activated twin variants are all relatively small among the six variants. It is shown that the activation of twin variants with a small SF is mainly attributed to local stress concentration. Therefore, the lower twinning fraction at 250 °C may also be ascribed to the activation of multiple slip systems during ET deformation and the occurrence of DRV, which effectively reduces the local stress concentration in the microstructure.

### 4.2. Dislocation Slip

**Figure 11:**
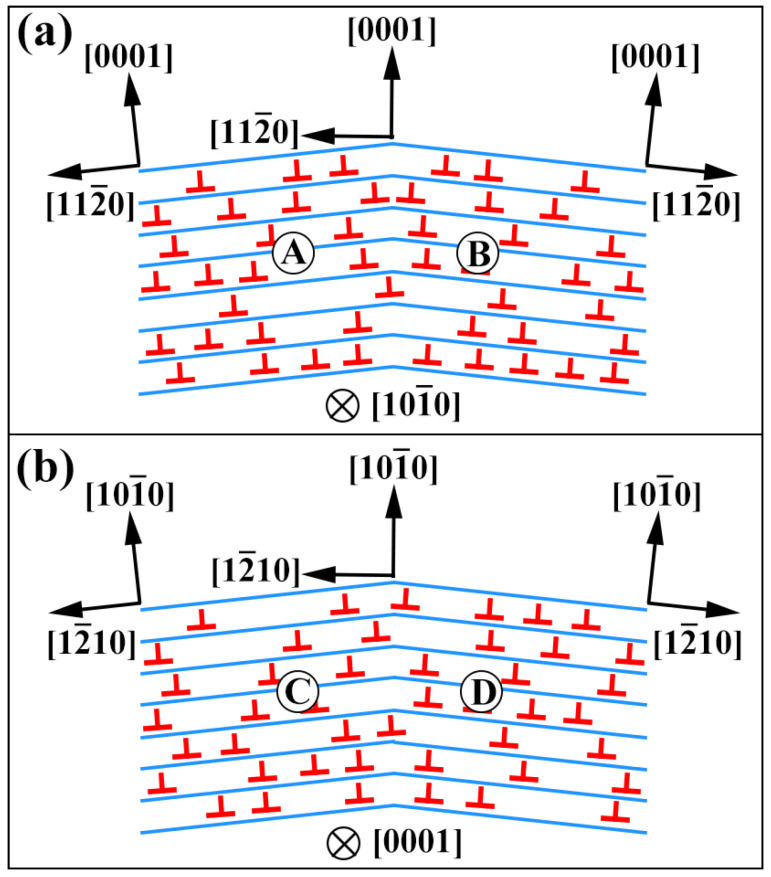
Schematic illustration of IGMA with (**a**) basal <a> slip and (**b**) prismatic <a> slip.

Figure 5 and Figure 6 show that the proportion of grains that undergo twinning during tensile deformation of the extruded AS51 alloy is low, both at RT and at 250 °C. In addition, the strain that can be directly accommodated by twinning is generally low. Meanwhile, LAGBs are abundantly present in Figure 5 and Figure 6. LAGBs are the result of intragranular dislocation slip, suggesting that dislocation slip is the principal deformation mode during the RT and ET deformation of the alloy [34]. To investigate the mechanism of dislocation slip during the tensile deformation of the extruded AS51 alloy, the in-grain misorientation axes (IGMA) analysis was performed on the microstructure after tensile deformation. IGMA can effectively determine the dominant intragranular slip mode and is widely used in the research of plastic deformation mechanisms of HCP alloys [35,36,37]. IGMA identifies the slip system generated within the grain by the preferred axis of lattice rotation within the grain, i.e., the Taylor axis. It is shown that the corresponding lattice rotation axes of basal <a> slip, prismatic <a> slip, and pyramidal II <c + a> slip in Mg alloys are <1–100>, <0001>, and <−1100>, respectively [38]. This can be understood by the schematic illustration shown in Figure 11. Figure 11a shows a basal <a> dislocation with a Burgers vector of 1/3 [11–20] on the (0001) plane generated in the bent single crystal. Thus, the deformed crystal bends near the line sense of the edge-type dislocation, i.e., [10–10] [39]. For the adjacent points A and B within the deformed crystal in Figure 11a, the EBSD-measured material point pairs should be rotated along [10–10]. Likewise, as shown in Figure 11b, when the prismatic <a> edge dislocation is activated, the rotation axis measured by EBSD should be (0001) for the adjacent points C and D within the deformed crystal.

**Figure 12:**
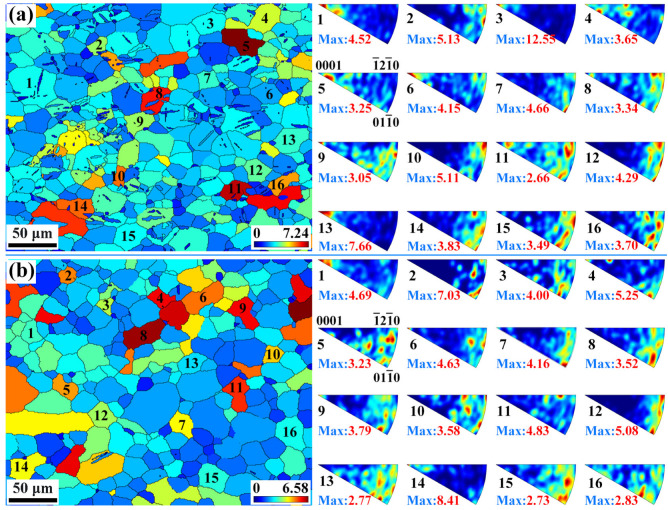
GOS maps of the extruded AS51 alloy at 15% strain and IGMA distribution of some grains: (**a**) RT, (**b**) 250 °C.

**Figure 13:**
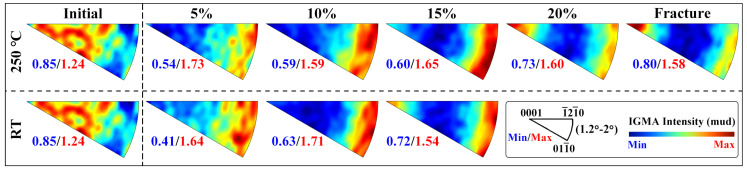
IGMA distribution for the extruded AS51 alloy at different strains at RT and 250 °C.

Figure 12a,b show the GOS maps of the extruded AS51 alloy at 15% strain at RT and 250 °C, as well as the IGMA distribution of some grains. GOS can usually reflect the strain level within the grain [40], and the microstructure under ET deformation conditions can still be found to have a lower strain level at the same strain through GOS maps. Based on the GOS maps, 16 grains with large GOS values under each of the two deformation conditions are randomly selected and the corresponding IGMA distribution maps are plotted. As shown in Figure 12a, for grains numbered 1, 3, 6, and 13, the IGMA distribution is concentrated at <0001>, suggesting that the dominant slip mode in these grains is prismatic <a>. The IGMA distribution of grain 7 shows a typical basal slip characteristic. Grains 10, 12, and 14 of IGMA can be found along <uvt0>, for RT deformation, which is also mainly attributed to basal slip, whereas the IGMA distributions of grains 8, 9, and 11 have no distinctive features. The GOS maps indicate high strain levels within them, and it is speculated that multiple slip modes may be activated simultaneously within these grains. The IGMA distribution of 16 grains in the microstructure at 15% strain at 250 °C is shown in Figure 12b, demonstrating that multiple slip modes are activated during the deformation. Figure 13 shows the overall IGMA distribution at different strains for the alloy at RT and 250 °C. It can be observed that the IGMA gradually changes from a uniform distribution before deformation to a <uvt0> distribution at 10% strain, which is mainly attributed to the large activation of basal slip at the initial stage of deformation. Although the SF of basal slip is the smallest in Table 1, the CRSS of the basal slip system is significantly lower than that of the non-basal slip system, so the basal slip is always the dominant deformation mode during the deformation of Mg alloy. When the strain is further increased to 15%, the IGMA maps at RT and 250 °C show <0001> distributions in addition to the obvious <uvt0> distributions, indicating that a certain amount of prismatic <a> slip already participated in the deformation at 15% strain. Under the deformation condition of 250 °C, as the strain continues to increase until fracture, the distribution of IGMA plots at <0001> is progressively evident, suggesting that prismatic <a> slip gradually evolves as the dominant deformation mode.

**Figure 14:**
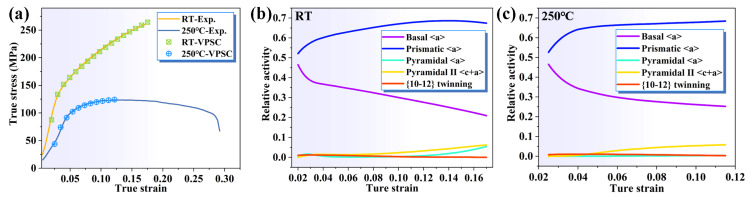
(**a**) True stress–strain curves and VPSC simulated curves for the extruded AS51 alloy at RT and 250 °C; the VPSC simulated relative activation of the different deformation modes during deformation at RT (**b**) and 250 °C (**c**).

To further investigate the mechanism of slip during the tensile deformation of the extruded AS51 alloy, the deformation of the alloy at RT and ET is simulated by VPSC. The VPSC framework is first reported by Lebensohn and Tomé, who use a self-consistent approach to describe the interactions between each grain and its local environment [41,42]. In the VPSC model, Voce hardening law is used to describe the hardening behavior of each slip system and twin in the following form:

(1) $$\tau_{c}^{s}=\tau_{0}^{s}+\left(\tau_{1}^{s}+\theta_{1}^{s}\Gamma \right)\left[1-exp\left(-\frac{\theta_{0}^{s}\Gamma }{\tau_{1}^{s}}\right)\right]$$

where $\tau_{c}^{s}$ represents the shear resistance of the deformation system s after strain Γ; $\tau_{0}^{s}$ denotes the initial CRSS. $\tau_{1}^{s}$, $\theta_{0}^{s}$ and $\theta_{1}^{s}$ are the hardening coefficients of the deformation system s, $\theta_{0}^{s}$ represents the initial hardening rate, $\theta_{1}^{s}$ represents the progressive hardening rate, and $\tau_{0}^{s}$ + $\tau_{1}^{s}$ denotes the CRSS of back extrapolation [43]. It is worth noting that the VPSC model only simulates the plastic deformation stage of the alloy. For the ET deformation process, VPSC only simulates the hardening stage of the plastic deformation of the alloy. The parameters applied for the VPSC simulations at RT and 250 °C are shown in Table 2. Figure 14a shows the simulated true stress–strain curves and the experimental true stress–strain curves. The simulated curves effectively match the experimental curves, indicating that the tensile deformation process of the alloy can be well simulated with the parameters in Table 2. The relative activation frequencies of different deformation modes during deformation at RT and 250 °C are shown in Figure 14b,c. At the early stage of deformation, basal slip and prismatic slip activation frequencies are comparable due to the low CRSS of basal <a> slip (Table 2) and prismatic <a> slip with high SF and moderate CRSS (Table 1 and Table 2). As the strain increases, the basal slip activation frequency gradually decreases and the prismatic slip gradually starts to dominate the deformation, which is consistent with the results of the IGMA analysis (Figure 13). For extension twinning, it is always at a lower frequency, which is consistent with the microstructure observations (Figure 5). Moreover, the <c + a> dislocation starts to be activated only at a certain strain due to having the largest CRSS, and the relative frequency is below 10%.

### 4.3. DRX Mechanism

**Figure 15:**
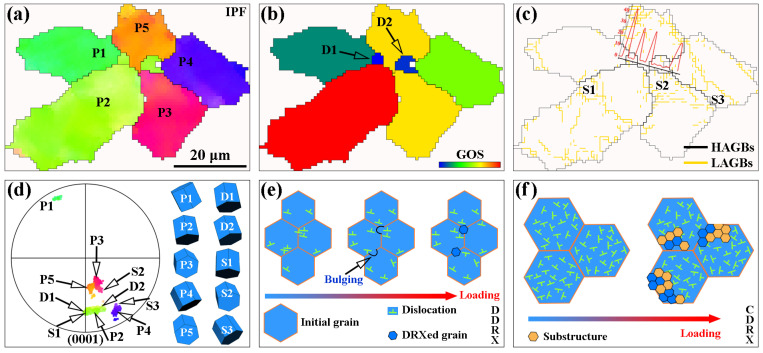
Typical DRX microstructure (R1 region in Figure 6d), (**a**) IPF map, (**b**) GOS map, (**c**) grain boundary map, (**d**) (0001) PF, (**e**) schematic of the DDRX mechanism, and (**f**) schematic of the CDRX mechanism.

Mg alloys usually exhibit better plastic deformability under ET deformation conditions than at RT. One of the reasons is that DRV and DRX can consume the accumulated dislocations during ET deformation, which can effectively relax the stress concentration near the grain boundaries, the second phase [11], and thus inhibit crack nucleation. DRV is usually difficult to characterize directly by experiments, while DRX can be directly observed by various characterization techniques, such as OM, EBSD, TEM, etc. As shown in Figure 15a, the IPF map of the R1 region in Figure 6d after enlargement, combined with the GOS map shown in Figure 15b, it can be determined that the grains D1 and D2 marked in the map are DRXed grains. DRXed grain D1 is at the trigonal grain boundaries of parent grains P1, P2, and P5. DRXed grain D2 is at the trigonal grain boundaries of parent grains P3, P4, and P5. Previous studies reported that DDRX is mainly nucleated at serrated grain boundaries and trigonal grain boundaries [44]. Therefore, the recrystallization process of DRXed grains D1 and D2 exhibits a typical DDRX characteristic. Figure 15e visualizes the process of DDRX, where the first dislocations accumulate at the original grain boundaries, and subsequently, numerous dislocations accumulate, leading to the formation of LAGBs protruding towards the adjacent grains [19]. Eventually, the LAGBs continuously absorb dislocations under strain and the substructure gradually transforms into DRXed grains. The red curve in Figure 15c shows the distribution of misorientation angles along the black lines through grains D1 and D2. It can be observed that the misorientation angles at the grain boundaries of DRXed grains D1 and D2 are greater than 30°, indicating that the LAGBs have been transformed into HAGBs. S1, S2, and S3 labeled in Figure 15c are substructures composed of LAGBs. The substructure formed within the grain also gradually forms DRXed grains under strain, as shown in Figure 15f, which is a typical continuous dynamic recrystallization (CDRX) mechanism [45]. Figure 15d shows the position of the parent grains, DRXed grains, and substructures in the (0001) PF. DRXed grains and substructures always have an orientation similar to that of the corresponding parent grain, indicating that recrystallization has a slight weakening effect on the texture. Overall, a few fine DRXed grains can be found in Figure 6e distributed near the initial HAGBs. Multiple LAGBs are distributed inside the grains and are not completely transformed into HAGBs, so the grain size is not significantly refined. In summary, the DRX mechanism is mainly DDRX with a low recrystallization fraction when the extruded AS51 alloy is deformed at 250 °C.

## 5. Conclusions

In this study, the tensile deformation process of the extruded AS51 alloy is investigated at RT and 250 °C. The major conclusions drawn are as follows:

(1) The TYS, UTS, and EL of the extruded AS51 alloy at RT are 122.7 MPa, 219.3 MPa, and 19.7%, respectively. The TYS, UTS, and EL at 250 °C are 89.2 MPa, 110.3 MPa, and 33.2%, respectively. Compared to RT deformation, TYS and UTS decreased by 27.3% and 49.7%, respectively, while EL improved by 68.5%.
(2) The extruded AS51 alloy displays continuous hardening behavior during the RT deformation of the alloy, and the late deformation at 250 °C leads to some softening due to the DRV and DRX, with the DRX mechanism being mainly DDRX and a low recrystallization fraction.
(3) The main deformation mechanism of the extruded AS51 alloy at RT and 250 °C is dislocation slip, with prismatic <a> slip as the dominant deformation mode, and no significant changes in grain size and texture type occur.
(4) The selection of twin variants is closely related to the local stress concentration. The relatively low activation of extension twins at 250 °C is partly attributed to the fact that the consumption of dislocations by DRV and DRX during deformation can effectively relax local stress concentrations.

## Figures and Tables

**Table 1:** SF of different deformation modes.

Conditions	Deformation Modes	5%	10%	15%	20%
RT	Basal <a>	0.23	0.19	0.20	
	Prismatic <a>	0.42	0.45	0.44	
	Pyramidal <a>	0.45	0.46	0.46	
	Pyramidal II <c + a>	0.43	0.44	0.43	
	{10–12} twinning	0.37	0.40	0.40	
250 °C	Basal <a>	0.24	0.21	0.21	0.20
	Prismatic <a>	0.43	0.44	0.44	0.44
	Pyramidal <a>	0.45	0.46	0.45	0.46
	Pyramidal II <c + a>	0.43	0.44	0.44	0.43
	{10–12} twinning	0.37	0.40	0.39	0.39

**Table 2:** Parameters used for VPSC simulations at RT and 250 °C.

Conditions	Deformation Modes	$\;\tau_{0}\;(\mathrm{MPa})$	$\tau_{1}\;(\mathrm{MPa})$	$\;\theta_{0}\;(\mathrm{MPa})$	$\theta_{1}\;(\mathrm{MPa})$
RT	Basal <a>	8	12	930	103
	Prismatic <a>	38	16	950	94
	Pyramidal <a>	47	52	800	51
	Pyramidal II <c + a>	82	67	950	42
	{10–12} twinning	16	0	0	0
250 °C	Basal <a>	4	28	400	0
	Prismatic <a>	19	25	300	0
	Pyramidal <a>	40	25	350	0
	Pyramidal II <c + a>	61	20	450	0
	{10–12} twinning	9	0	0	0

## Data Availability

The data presented in this study are available upon request from the corresponding author.
